# Gonadotropin-releasing hormone (GnRH) stimulation test before and after GnRH analogue treatment in central precocious puberty; can GnRH test simplify adequately?

**DOI:** 10.1186/1687-9856-2013-S1-P73

**Published:** 2013-10-03

**Authors:** You Jean Yang, Min Sun Kim, Pyoung Han Hwang, Dae-Yeol Lee

**Affiliations:** 1 Department of Pediatrics, Chonbuk National University Medical School, Jeonju, Korea

## Purpose

Gonadotropin-releasing hormone (GnRH) stimulation test is the gold standard to document premature activation of the hypothlamic-pituitary-gonadal axis in early puberty. However, this test is time-consuming, costly and uncomfortable for the patients. The aim of this study was to investigate to simplify the GnRH stimulation test in the assessment of pubertal activation and suppression.

## Methods

We identified 72 girls diagnosed with central precocious puberty, and they were treated with GnRH analogue(GnRHa) to suppress pubertal progression from 1 January 2011 to 31 January 2012. GnRH stimulation tests were done before and 4 weeks after the third dose of the GnRH analogue. Information on clinical manifestations and laboratory data were obtained by reviewing medical records. All variables were expressed as mean +/- SD, and a *p* value of <0.05 was considered statistically significant.

## Results

1) Before GnRHa treatment, the mean luteinizing hormone (LH) level was higher at the 30^th^ minutes (18.17 IU/L±16.77) of the test in comparison to the basal level (0.35 IU/L ± 0.60), the 15^th^ , 60^th^, 90^th^ and 120^th^ minutes (p<0.001). However, there was no significant difference in the LH level between the 30^th^ and 45^th^ minutes of the test. Among 72 patients, 49 girls (68.1%) were showed the peak LH level at 30^th^ minutes and the others were showed the peak LH level at 45^th^ minutes (33.3%), and 60^th^ minutes (6.9%).

2) After GnRHa treatment, 62 patients (86.1%) were suppressed (peak LH level < 2 IU/L), and 10 (13.9%) were inadequately suppressed. The LH level was higher at the 30^th^ minutes (0.91 IU/L±1.14) of the test in comparison to the basal level (0.26 IU/L ± 0.55), the 45^th^ minutes (0.78 IU/L±1.12) and 60^th^ minutes (0.71 IU/L±1.08)(*p*<0.001). But the LH level was no significant difference between 15^th^ and 30^th^ minutes.

3) After GnRHa treatment, the AUC in ROC analysis was greatest at the 30^th^ minutes, and sensitivity and specificity of the 30^th^ minutes samples were 90.0% and 100.0%. The basal LH level of the test was showed low sensitivity (25.0%).

## Conclusion

It is adequate to check the LH level at 30^th^ and/or 45^th^ minutes of the GnRH test for diagnosis of CPP and 30^th^ minutes for assessment of pubertal suppression. Therefore we suggest that the simplified GnRH test is sufficient to evaluation of pubertal activation and suppression.

